# Maternal nicotine intoxication before pregnancy induces depressive‐ and anxiety‐like behaviors as well as cognitive deficits in male offspring and correlates with neurobiological changes

**DOI:** 10.1002/brb3.3052

**Published:** 2023-05-09

**Authors:** Linglong Deng, Qing Wang, Ying Lou

**Affiliations:** ^1^ Department of Nursing, Nanjing Vocational Health College Nanjing China; ^2^ Department of Pharmacy, Nanjing Mochou Vocational School Nanjing China

**Keywords:** anxiety, depression, nicotine, offspring

## Abstract

**Introduction:**

Maternal nicotine use has been suggested to affect the behavior of children and is linked to changes in neurological systems; however, the specific mechanism is yet to be understood.

**Methods:**

Mice were used to establish a maternal nicotine intoxication model. At postnatal day 60 (adolescent stage), male offspring were tested for behavioral tasks including sucrose preference, open field, elevated plus maze, light/dark box, object recognition, Morris water maze (MWM), and forced swimming. Enzyme‐linked immunoassays were used to measure plasma concentrations of neurotransmitters including norepinephrine, dopamine, serotonin, and corticosterone. Serotonin transporter (*Sert*), brain‐derived neurotrophic factor (*Bdnf*), cAMP response element binding protein (*Creb*), and phosphorylated (*p*)*Creb* mRNA levels were measured using quantitative real‐time polymerase chain reaction.

**Results:**

Male offspring of nicotine‐intoxicated dams had significantly reduced sucrose preference, mobility time in the forced swimming test, and locomotor and exploratory activities. Offspring in the maternal nicotine intoxication group showed increased signs of depressive‐ and anxiety‐like behavior. Recognition memory in the MWM was compromised in these animals. The hippocampal and prefrontal cortical regions showed significant changes in *Bdnf*, *pCreb*, and *Sert* gene expression, whereas *CREB* mRNA levels were unaffected. Moreover, compared to the controls, neurogenesis and neuronal viability were also reduced in these animals.

**Conclusion:**

Prenatal nicotine exposure might affect the hypothalamic–pituitary–adrenal axis and reduce neurogenesis, potentially leading to depressive‐like behaviors and cognitive deficiencies in male offspring.

## 1 INTRODUCTION

Tobacco smoke affects not only smokers, but also more than 40% of children, 33% of nonsmoking males, and 35% of nonsmoking women (Hernández‐Martínez et al., 2017). Smoking cigarettes during pregnancy has been linked to a variety of negative effects in both mothers and children (Bruin et al., 2010). Premature delivery, membrane rupture, intrauterine developmental delay, and sudden infant death syndrome are all exacerbated by maternal exposure to cigarette smoke, whether passive or active (Holbrook, 2016). Long‐term and delayed detrimental effects of nicotine on the cognitive development of children have been linked to maternal smoking during pregnancy (Balázs et al., 2018). Furthermore, human milk contains nicotine, which is absorbed by infants and is linked to behavioral irritability and anxiety (Karimiankakolaki et al., 2019). Limiting or eliminating cigarette use during pregnancy may lessen the negative effects on the developing baby (Raatikainen et al., 2007).

Previously, it was reported that maternal nicotine exposure results in depressive‐ and anxiety‐like behavior in offspring; however, the exact underlying mechanism is yet to be understood. In this study, we hypothesized that nicotine‐mediated effects on the hypothalamic–pituitary–adrenal (HPA) axis are involved in brain neurogenesis in offspring. In terms of background, the HPA axis affects a person's stress response (Haj‐Mirzaian et al., 2017; Hinds & Sanchez, 2022). This axis controls hormone synthesis and release, making it crucial for the ability to deal with adversity. Most individuals with depression have a hyperactive HPA axis (Haj‐Mirzaian et al., 2021; Pariante & Lightman, 2008). Corticosteroids have been shown to affect emotional behaviors and cognition in animals and humans in a complex manner (Bos et al., 2012). Furthermore, the serotonergic and adrenergic systems have been linked to hippocampal‐dependent memory because of their roles in modulating functional neural circuits in the brain (Katsouri et al., 2013). In addition, studies have shown that the hippocampus, hypothalamus, and prefrontal cortex play primary roles in the neuroendocrine control of feeding, emotion, and metabolism in adulthood, and that these regions are involved in the stress response and are the areas most relevant to depression (Berry et al., 2022). Furthermore, monoaminergic signaling pathways primarily function via G‐proteins, which, in turn, activate the pathway that involves adenylyl cyclase, protein kinase A, and the transcription factor cAMP response element binding protein (CREB). Multiple target genes implicated in the pathophysiology of depression are regulated by phosphorylated (p)CREB (Blendy, 2006). The hippocampus, hypothalamus, and prefrontal cortex have been studied separately and in conjunction with the HPA axis, serotonergic and adrenergic systems, and monoaminergic signaling pathways to examine their roles in depression.

Our hypotheses for this study were as follows: (1) prenatal exposure to nicotine results in behavioral abnormalities in male offspring, and (2) these behavioral abnormalities are linked to the HPA axis, monoaminergic system, and disruption of signal transduction pathways. To examine our hypotheses, we subjected pregnant mice to nicotine intoxication. The pups born to these mothers then underwent behavioral assessments including the open field test (OFT), sucrose preference test (SPT), elevated plus maze (EPM) test, light/dark box (LDB), forced swim test (FST), object recognition test (ORT), and Morris water maze (MWM) test. Brain region‐specific neurotransmitter levels were measured, as well as the mRNA expression of pCREB, serotonin transporter (SERT), and brain‐derived neurotrophic factor (BDNF), and the relationship between behavioral and molecular alterations was analyzed. Finally, we evaluated neurogenesis and neural viability of the offspring brains to clarify the results.

## 2 MATERIALS AND METHODS

### 2.1 Animals

We used adult C57BL/6 mice for breeding. Mice had free access to food and water and were kept in a vivarium with a conventional 12 h light–dark cycle (lights on at 7.00 a.m.). The temperature and humidity were maintained at 22°C and 50%, respectively. The Institutional Animal Care and Use Committee at Nanjing Vocational Health College approved all procedures used in this study. These procedures were carried out in accordance with the principles outlined in the Guide for the Care and Use of Laboratory Animals (NIH publication no. 8023, updated 1978).

### 2.2 Maternal nicotine intoxication

A total of 10 female mice were administered nicotine (Sigma–Aldrich, St. Louis, MO, USA; N‐3876; 200 μg/mL) in water sweetened with 1% saccharin, beginning 2 weeks before mating and continuing through weaning of the pups. Ten female control mice were given saccharin‐sweetened water for comparisons, representing the experimental control group. Both groups were monitored to make sure their water intake was increased. All female mice drank nicotine or control water for 2 weeks before being coupled with male mice in a 3:1 ratio until they had their litters. All female mice in both experimental groups were reproduced. Offspring were assigned randomly to experimental groups on postnatal day 21 (P21).

### 2.3 Open field test

At P35, testing began in an open field. An overhead camera and the behavior tracking program, EthoVision XT 5.1 (Noldus Information Technology, The Netherlands), were used to monitor locomotor activity. After acclimating each mouse to the behavioral recording room for 60 min and the arena for 10 min over the course of 3 days, each animal was placed gently in the center of a 30 × 30 × 35 cm Plexiglas transparent chamber and allowed to roam for 1 h. The region 7.5 cm from the wall was termed the margin. The remainder was the core area. Between experiments, each chamber was sanitized with 10% ethanol to ensure that no lingering odors would affect the results. The distance that the mouse moved throughout the trial was measured in centimeters from its starting position. Periods of immobility (i.e., when the EthoVision recorded no detectable forward or backward motion on the part of the animal) were measured in seconds. A mouse was considered stationary if the total area represented by its pixels moved by less than 20% from one three‐frame sample to another. It is unlikely that a mouse rearing its young or getting groomed would be recognized as motionless.

### 2.4 Forced swim test

The FST technique was developed by Porsolt et al. (1979). In this test, individual mice were confined in a cylindrical flask (with a diameter of 10 cm and a height of 25 cm) with 19 cm of water maintained at 23 ± 1°C. Mice were given 6 min to swim in this pool; the final 4 min of the test were dedicated to recording the length of time the animals were still. When a mouse stopped struggling and floated without moving, other than maintaining its head above water, we deemed it immobile (Haj‐Mirzaian et al., 2015).

### 2.5 EPM test

Mouse anxiety levels were evaluated using the EPM test. The mice were tested in EPM equipment with two 45 × 5 cm lanes arranged in a plus form. There was a square open platform 5 × 5 cm in the middle where the two planes met. To allow the mice to become accustomed to their new surroundings and lower their stress levels, they were brought into the behavioral lab approximately 3 h before the experiment began. Sunlight (150−200 lx) was used during the experiment. The mice were taken out of their cage and positioned headfirst at the intersection of the open and closed arms, with their backs to the experimenter and their ears facing the open arms. The rodents roamed freely, and the camera recorded their movements that were then analyzed using Smart v2.5.21. Between tests, 75% ethanol was used to clean the maze.

### 2.6 LDB test

The LDB was constructed from two separate sections of Plexiglas. The larger (25 × 25 × 30 cm), brighter part was lit by a 75‐W white‐light bulb (approximately 200 lx). The smaller, darker area was 18 × 25 × 30 cm and was lit by a single 40‐W red light bulb (approximately 30 lx). The bottom of the box had 45 cm distance from both bulbs. A wall with a 6.5 × 6.5 cm entryway divided the two rooms. Each time an experiment was conducted, the animal was positioned in the middle of the bright room with its back facing the dividing wall. When a mouse passed through the opening and planted all four paws in the opposite room, an entrance was counted. The number of times a subject entered and exited the lit compartment and the total amount of time spent in the chamber served as measures of their activity and anxiety levels. Behaviors including rearing, climbing walls, and grooming were also tracked for frequency.

### 2.7 Object recognition test

The offspring were subjected to open field testing for four consecutive days according to the ORT technique reported in the literature (Wei et al., 2018). The animals were first introduced to the setup on days 1 and 2 at 10:00 a.m. when they were given free reign of the enclosure. The box test apparatus was 40 × 40 × 40 cm. On the third day of training, the animals were shown two identical metal cans positioned against one wall of the box for 5 min. Each mouse was left in the arena and placed in the center of the opposing wall with its back facing the two cans. The amount of time spent investigating each object was recorded using SuperMaze video tracking technology. The amount of time the animal spent with its nose within a 2 × 2 cm region surrounding the cans was recorded. Following the hour‐long training session, animals were returned to their original cages. The arena was then changed to feature two new items before the animal was again released. One was a new metal, glass, or hard plastic object, whereas the other was the same as the metal cans used in the training session but had never been used before. Over the course of 5 min, the amount of time spent investigating each item was recorded. The mice were tested once more with the familiar object and a novel object on day 4 for 5 min. All animals were given the same number of novel objects, but the order in which they were presented was randomized. Because of the importance of avoiding olfactory signals, each item in this experiment was used only once. On days 3 and 4, the unknown objects were always different from the familiar ones in terms of material, color, size, and form. At the conclusion of each experiment, 75% ethanol was used to disinfect the apparatus and the test area. The recognition index (RI) is the primary index for analyzing memory, and is defined as the ratio of time spent examining the novel item to the total time spent exploring novel and familiar things (or the response to novelty). With an RI above 50%, the novel item is explored for a longer period of time than the familiar one, and vice versa with an RI below 50%. When the RI is exactly 50%, there was no clear preference.

### 2.8 Morris water maze

Mice in each group were acclimated to the water maze laboratory before the experiment began. The MWM is large, measuring 120 cm in circumference and 50 cm in depth. The pool was divided into four sections, each of which had a transparent glass platform measuring 9 cm in diameter and 11 cm in height. Water at 25°C was poured into the pool after the platform was set up (1 cm higher than the platform). Then, to prevent the mice from seeing the platform, milk powder was added to the water and mixed in thoroughly. In addition, an infrared smart camera was placed above the pool. To aid in imaging during the water maze experiment, a red dye was applied to the head of each mouse before beginning the test. There are three basic types of analysis in the MWM test: positional navigation (for acquired training), space exploration (for exploratory training), and visual platform (for testing).

### 2.9 Sucrose preference test

Two bottles of sucrose solution (1%, w/v) were used to acclimatize rats. After 24 h, one of the sucrose solutions was replaced with water. On the third day, the rats were fasted for 23 h without access to water. For the last hour of the experiment, they had free access to two bottles of solution that were weighed beforehand. A scale was used to determine the amount of sucrose and plain water removed from each bottle. To determine the sucrose preference rate, we used the following formula: sucrose preference rate = sucrose consumption (g)/[water consumption (g) + sucrose consumption (g)].

### 2.10 Conditioned place preference

Researchers employed this measure as a physical indication of ethanol sensitization (Libarino‐Santos et al., 2020). The preference apparatus was constructed of wood and had two equal‐sized square bases (15 × 15 × 30 cm). Two distinct compartments were created using different visual and tactile texture cues: one compartment was painted black inside and its floor smooth, and the other compartment was painted white inside and its floor textured. An objective place conditioning method was used. In this setup, medication delivery was randomly matched with either compartment, and the mice did not exhibit a clear preference for either of the compartments throughout the preconditioning test. The conditioned place preference (CPP) paradigm covered 9 days and consisted of three stages: familiarization, preconditioning, and conditioning/postconditioning. The experiments were conducted between 10.00 a.m. and 2.00 p.m. Each mouse was given 10 min of unrestricted access to the device on days 1 (familiarization) and 2 (preconditioning).

The conditioning phase included six 40‐min sessions spread across 6 days. A detachable sheet was used to create an isolation chamber, and the animals were confined to their designated areas every other day. In this study, we used intraperitoneal administration of ethanol as the drug at doses of 0.5 and 5 mg/kg of body weight. The mice were administered ethanol on days 1, 3, and 5, and the vehicle on days 2, 4, and 6. After receiving ethanol or vehicle during conditioning sessions, or during the postconditioning test session, the time spent in both ethanol‐paired and in the vehicle‐paired compartments of each animal was measured by dividing the floor of each compartment into four equal‐sized squares (7.5 × 7.5 cm) and recording the number of mouse entrances to each of the squares by an unbiased observer. We recorded time spent in both compartments for each animal and used it to calculate the CPP score, which was defined as the difference between the times spent in the ethanol‐paired and in the vehicle‐paired compartments. The average of these values was used in the statistical analyses.

### 2.11 Acquisition of brain tissue samples

After the behavioral trial was completed, CO_2_ euthanasia was administered. Following euthanasia, the brain was extracted by severing the parietal and optic nerves. For the quantitative real‐time polymerase chain reaction (qRT‐PCR) gene expression analyses, the entire brain was removed and placed on ice. Then, the hippocampal formation, hypothalamus, left hippocampus, and left prefrontal cortex were carefully removed, placed in 4% polyformaldehyde solution for fixation or into liquid nitrogen for quick freezing, and then transferred to a −80°C freezer. Polyformaldehyde‐fixed samples were cut continuously using a microtome (5 μm thick) for analysis.

### 2.12 BrdU and doublecortin immunofluorescence staining for evaluating mouse hippocampal neurogenesis and neural viability

For BrdU staining, 1% Triton X‐100 and 0.5% Tween 20 in phosphate‐buffered saline were used to permeabilize the slices. Then, 1 N HCl was added for 10 min followed by 2 N HCl for 10 min. Both incubations were performed at room temperature. This was followed by incubating for 20 min at 37°C to denature the DNA. Blocking utilized with 5% goat serum (Beyotime Biotechnology, Haimen, China, C0265) for 1 h, then overnight incubation with anti‐BrdU and anti‐doublecortin (DCX) antibodies at 4°C, followed by washing with borate buffer (0.1 M) and Tris‐buffered saline. Slices were finally washed with phosphate‐buffered saline and incubated at room temperature for 90 min with secondary goat anti‐rat IgG H+L. Nuclei were stained with 4′,6‐diamidino‐2‐phenylindol (1:2000; Beyotime Biotechnology, 1002). Antifade mounting medium (Beyotime Biotechnology, P0126) was used to mount the slices, which were then kept at 4°C in the dark until image acquisition and analysis. It should be noted that, for evaluating neural viability, we extracted neural stem cells from the hippocampus of the offspring from both normal and maternal nicotine‐intoxicated mice, and cells were cultured as previously described.

### 2.13 qRT‐PCR assessment

TRIzol reagent (Invitrogen, Carlsbad, CA, USA) was used to extract total RNA from the hippocampal and right prefrontal cortex tissues, and a ReverTra Ace qPCR RT Kit was used to reverse transcribe the RNA into cDNA (Toyobo, Osaka, Japan). The Ultra‐SYBR Mixture was used for qRT‐PCR (CWBIO, Beijing, China). *Bdnf*, *Creb*, *pCreb*, and *Sert* mRNA levels were evaluated in the samples using the qRT‐PCR. All primers used are listed in Table 1.

**TABLE 1:** Primer sequences used in this study

Gene	Primer sequence 5′−3′
BDNF	Forward 5′‐ TCATACTTCGGTTGCATGAAGG‐3′
	Reverse 5′‐AGACCTCTCGAACCTGCCC‐3′
SERT	Forward 5′‐ CAAGCCCTGCTCCGAATCTC‐3′
	Reverse 5′‐GCTGGAGTGTGTTGTGGATAA‐3′
CREB	Forward 5′‐ AGGATCTTCTGCCGTCTTGAT‐3′
	Reverse 5′‐GCGCAGCCTTCAGTCTCAT‐3′
p‐CREB	Forward 5′‐ CCGTGTTTGATCGGCAGGAC‐3′
	Reverse 5′‐CGCCGACCATAATGGAGA‐3′

### 2.14 Evaluation of corticosterone, adrenocorticotropic hormone, and monoamine neurotransmitter levels

Corticosterone, adrenocorticotropic hormone (ACTH), and monoamine neurotransmitter levels were measured in brain and plasma samples. Plasma was extracted from the blood samples by centrifugation at 1250 × *g* (15 min, 4°C). Brain tissue (left hippocampus and left prefrontal cortex) was homogenized before being transported for analysis. Enzyme‐linked immunoassay kits were used to measure corticosterone, ACTH, and monoamine neurotransmitter concentrations according to the manufacturer's instructions.

### 2.15 Statistical analyses

Prism 9 software (GraphPad, San Diego, CA, USA) was used for statistical analyses. A *p*‐value of <.05 was considered statistically significant. One‐way ANOVAs were used to evaluate the differences in means between groups because all data followed a normal distribution. The study's sample size was calculated using G*Power version 3 software (https://www.psychologie.hhu.de/arbeitsgruppen/allgemeine‐psychologie‐und‐arbeitspsychologie/gpower), with a power of 0.8 and a significance level of *α* = .05.

## 3 RESULTS

### 3.1 Effects of nicotine intoxication on maternal and adult offspring physiological and behavioral development

**FIGURE 1:**
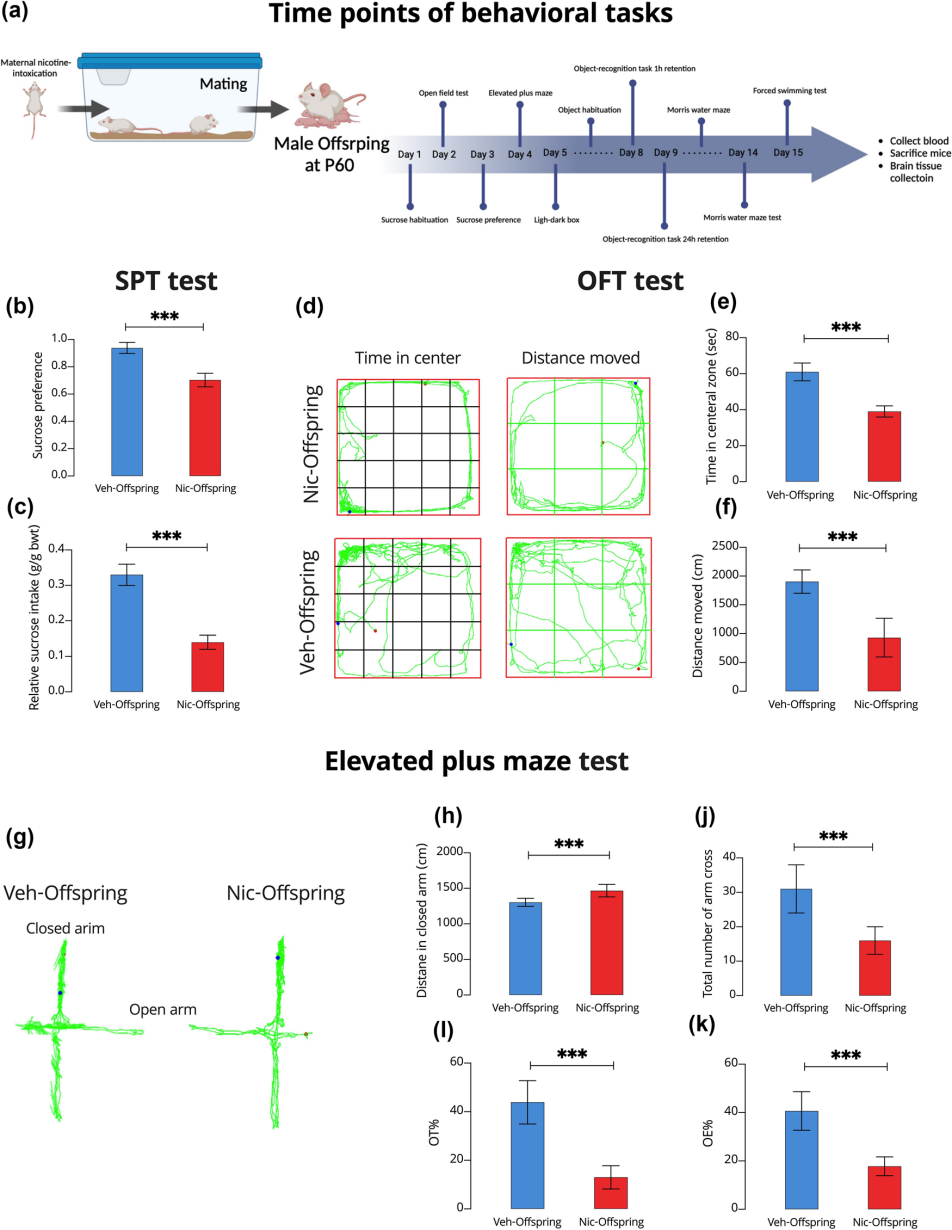
The effect of maternal nicotine intoxication on the sucrose preference test (SPT), open field test (OFT), and elevated plus maze (EPM) test. (A) Timeline of the study design. (B) SPT results. (C) Relative sucrose intake. (D) Schematic of mouse movements in the OFT using EthoVision. (E) Total time spent in the center zone during the OFT. (F) Total distance moved during the OFT. (G) Schematic of mouse movements in the EPM test using EthoVision. (H) Distance moved in the closed arm of the EPM. (I) Percentage of time in the open arm (OT%) of the EPM. (J) Total number of arm crosses. (K) OT% of the EPM. All data are presented as means ± SD. One‐way analyses of variance were used to evaluate the differences between offspring from control (veh‐offspring) and nicotine‐intoxicated (nic‐offspring) dams. ***p < .001.

The timeline of the behavioral tasks is shown in Figure 1A. In this study, we observed that pregestational exposure to nicotine altered behavioral tasks in male offspring. Specifically, the male offspring of nicotine‐intoxicated maternal mice (nic‐offspring) showed abnormal locomotor activity in the SPT (Figure 1B,C), OFT (Figure 1D–F), and EPM (Figure 1G–K). Although all animals in the SPT showed a preference for the sucrose solution over water, nic‐offspring had lower sucrose intake (sucrose preference index and relative sucrose intake) compared with controls (veh‐offspring) (*p* < .001; Figure 1B,C). Nic‐offspring covered less ground and spent less time in the central area than the veh‐offspring (*p* < .001; Figure 1E). The overall distance moved in the OFT was greater in the veh‐offspring than in nic‐offspring (*p* < .001; Figure 1F). In the EPM test, the total distance moved in the closed arm was significantly greater in the nic‐offspring than in the veh‐offspring (*p* < .001; Figure 1H). In addition, the total number of arm crossings in the veh‐offspring group was significantly higher than that in the nic‐offspring group (*p* < .001; Figure 1J). Nic‐offspring also spent less time in the open arm (OT%) and entered the arm (OE%) less often than did their control counterparts (*p* < .001; Figures 1I and 1K, respectively).

**FIGURE 2:**
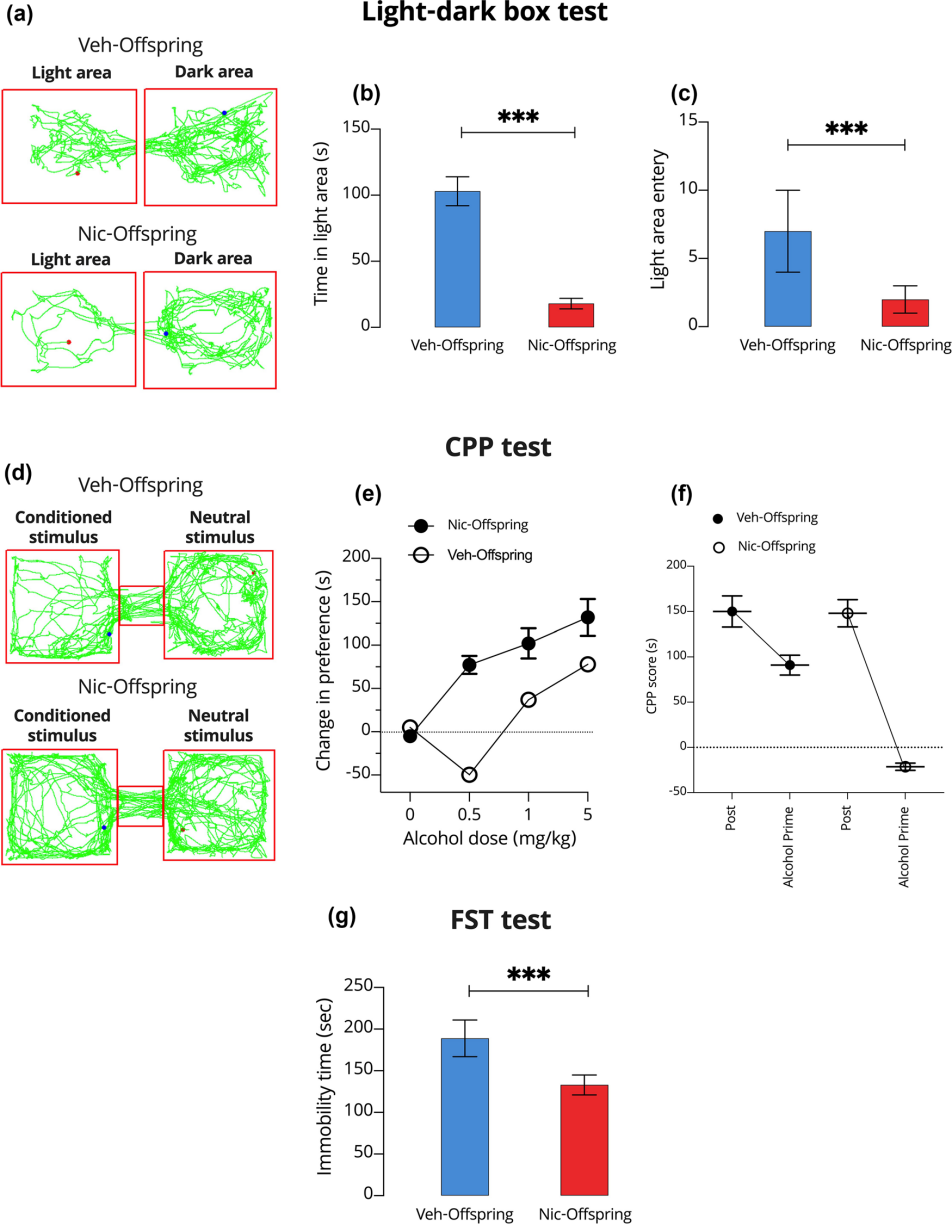
The effect of maternal nicotine intoxication on the light‐dark box (LDB) test, conditioned place preference (CPP) test, and forced swimming test (FST). (A) Schematic of mouse movements in the LDB using EthoVision. (B) Time spent in the light area. (C) Number of entries to the light area. (D) Schematic of mouse movements in the CPP test using EthoVision. (E) Change in preferences after injection of different doses of ethanol. (F) Evaluation the CPP score pre‐ and post‐ethanol. (G) Immobility time of the FST. All data are presented as means ± SD. One‐way analyses of variance were used to evaluate differences between offspring from control (veh‐offspring) and nicotine‐intoxicated (nic‐offspring) dams. ***p < .001.

In the LDB test, maternal nicotine intoxication resulted in abnormal behavior of the offspring (Figure 2A–C). Pregestational nicotine intoxication was associated with reduced staying in the light box, light‐side entry rates, and light‐side movement in the offspring compared to controls (*p* < .001; Figures 2B and 2C, respectively). On the other hand, in the CPP test, mice in the nic‐offspring group had a higher and dose‐dependent preference to stay in the chamber after receiving ethanol compared to the control group (*p* < .001; Figure 2E). In addition, we observed a significant decrease in the CPP score in the ethanol pre‐ to posttest in nic‐offspring compared to the control group (*p* < .001; Figure 2F). In the FST, we observed enhanced depression‐like behavior in the nic‐offspring than in veh‐offspring (*p* < .001; Figure 2G).

**FIGURE 3:**
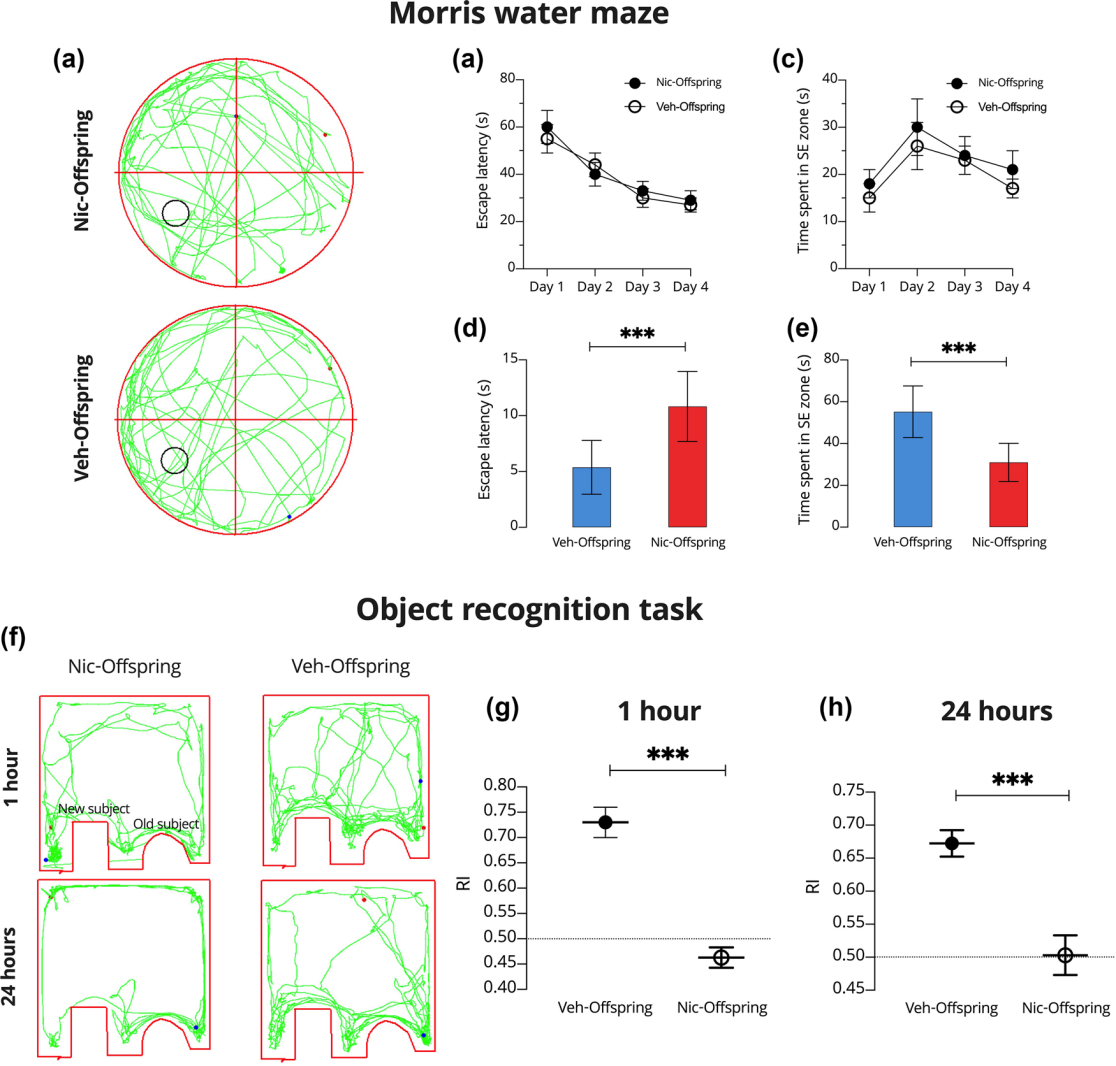
The effect of maternal nicotine intoxication on the Morris water maze (MWM) and object recognition test (ORT). (A) Schematic of mouse movements in the MWM using EthoVision. (B) Training day escape latency. (C) Time spent in SE (standard error) (target zone) in training sessions. (D) Test day escape latency. (E) Time spent in SE on the test day. (F) Schematic of mouse movements in the ORT using EthoVision. (G) Results of the ORT using the recognition index after 1 h. (H) Results of the ORT using the recognition index after 24 h. All data are presented as means ± SD. One‐way repeated measure ANOVAs were used to evaluate differences between offspring from control (veh‐offspring) and nicotine‐intoxicated (nic‐offspring) dams. ***p < .001.

In the MWM test, the nic‐offspring exhibited abnormal learning and memory behavior (Figure 3A–E). Although pregestational nicotine exposure had no significant influence on escape latency or time spent in the SE (standard error) zone during the acquisition phase of the MWM task (*p* > .05; Figure 3B,C), in the test phase, nic‐offspring had a significantly longer escape latency than veh‐offspring (*p* < .001; Figure 3D). This suggests that pregestational nicotine intoxication caused a significant impairment in the retention phase. In addition, veh‐offspring swam significantly shorter distances, spent significantly less time in the SE zone, and crossed the platform at a significantly lower rate than nic‐offspring (*p* < .001; Figure 3E). Finally, nic‐offspring demonstrated recognition memory deficits in a novel object recognition test, with the discrimination index being significantly lower than controls for both the 1‐h (*p* < .001; Figure 3G) and 24‐h (*p* < .001; Figure 3H) memory retention tasks.

### 3.2 Effect of maternal nicotine intoxication on HPA responsiveness

**FIGURE 4:**
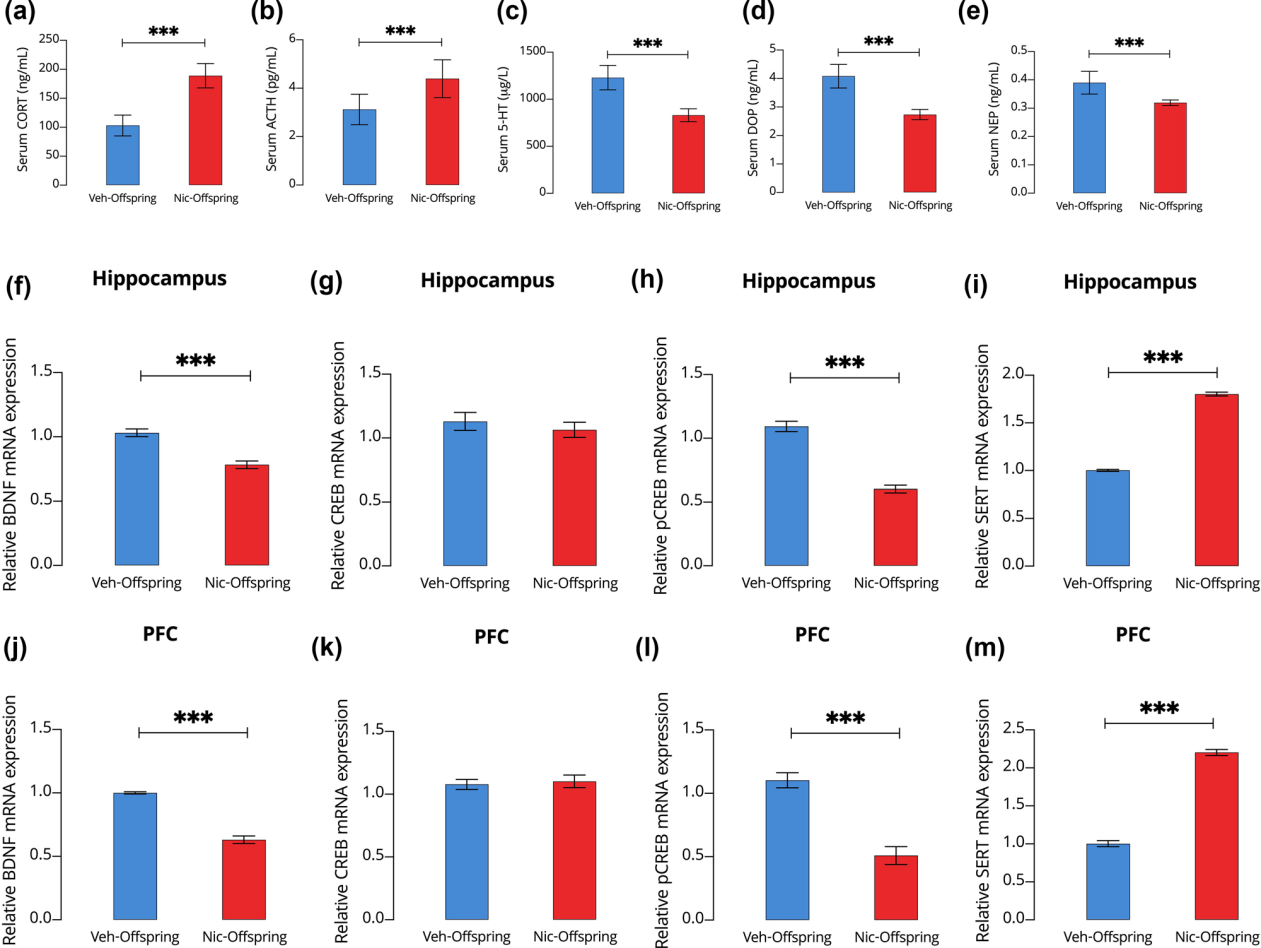
The effect of maternal nicotine intoxication on plasma levels of corticosterone (CORT), adrenocorticotropic hormone (ACTH), serotonin (5‐HT), norepinephrine (NE), and dopamine (DOP); hippocampal and prefrontal cortex (PFC) mRNA levels of brain‐derived neurotrophic factor (BDNF), cAMP response element binding protein (CREB), phosphorylated (p)CREB, and serotonin transporter (SERT); and conditioned place preference (CPP) and forced swimming test (FST) results. One‐way ANOVAs were used to evaluate the differences of serum CORT (A), ACTH (B), 5‐HT (C), DOP (D), and NE (E), as well as hippocampus and PFC levels of BDNF (F and J, respectively), CREB (G and K, respectively), pCREB (H and L, respectively), and SERT (I and M, respectively) between offspring from control (veh‐offspring) and nicotine‐intoxicated (nic‐offspring) dams. All data are presented as means ± SD. ***p < .001.

Plasma corticosterone and ACTH levels in the nic‐offspring were substantially higher (*p* < .001; Figures 4A and 4B, respectively) than in the veh‐offspring. In line with the previous result, serotonin levels in the plasma of nic‐offspring were significantly lower than those in veh‐offspring (*p* < .001; Figure 4C), suggesting that the monoaminergic system was involved in the effects of prenatal maternal nicotine intoxication. Furthermore, nic‐offspring had lower plasma levels of dopamine and norepinephrine than the control group (*p* < .001; Figures 4D and 4E, respectively). These results suggest that maternal nicotine intoxication could result in altered HPA axis responsiveness, and subsequently lead to depression‐ and anxiety‐like behaviors in offspring, as well as cognitive behavioral impairment.

### 3.3 Maternal nicotine intoxication resulted in abnormal expression of BDNF, pCREB, and SERT mRNAs in the hippocampus and prefrontal cortex of offspring

We explored the mRNA levels of *Bdnf*, *Creb*, *pCreb*, and *Sert* to evaluate the effect of maternal nicotine intoxication on brain neurogenesis factors in the offspring. Compared to controls, nic‐offspring had significantly lower *Bdnf* and *pCreb* mRNA levels in the hippocampus (*p* < .001; Figures 4F and 4H, respectively) and prefrontal cortex (*p* < .001; Figures 4J and 4L, respectively). The relative mRNA expression of *Creb* in the hippocampus and prefrontal cortex of nic‐offspring and controls did not differ significantly (*p* > .05; Figures 4G and 4K, respectively). Finally, we observed that the relative expression of *Sert* in the hippocampus and prefrontal cortex of nic‐offspring was significantly higher than that in controls (*p* < .001; Figures 4I and 4M, respectively).

### 3.4 Male offspring of nicotine‐exposed dams had lower neuronal regeneration and viability

**FIGURE 5:**
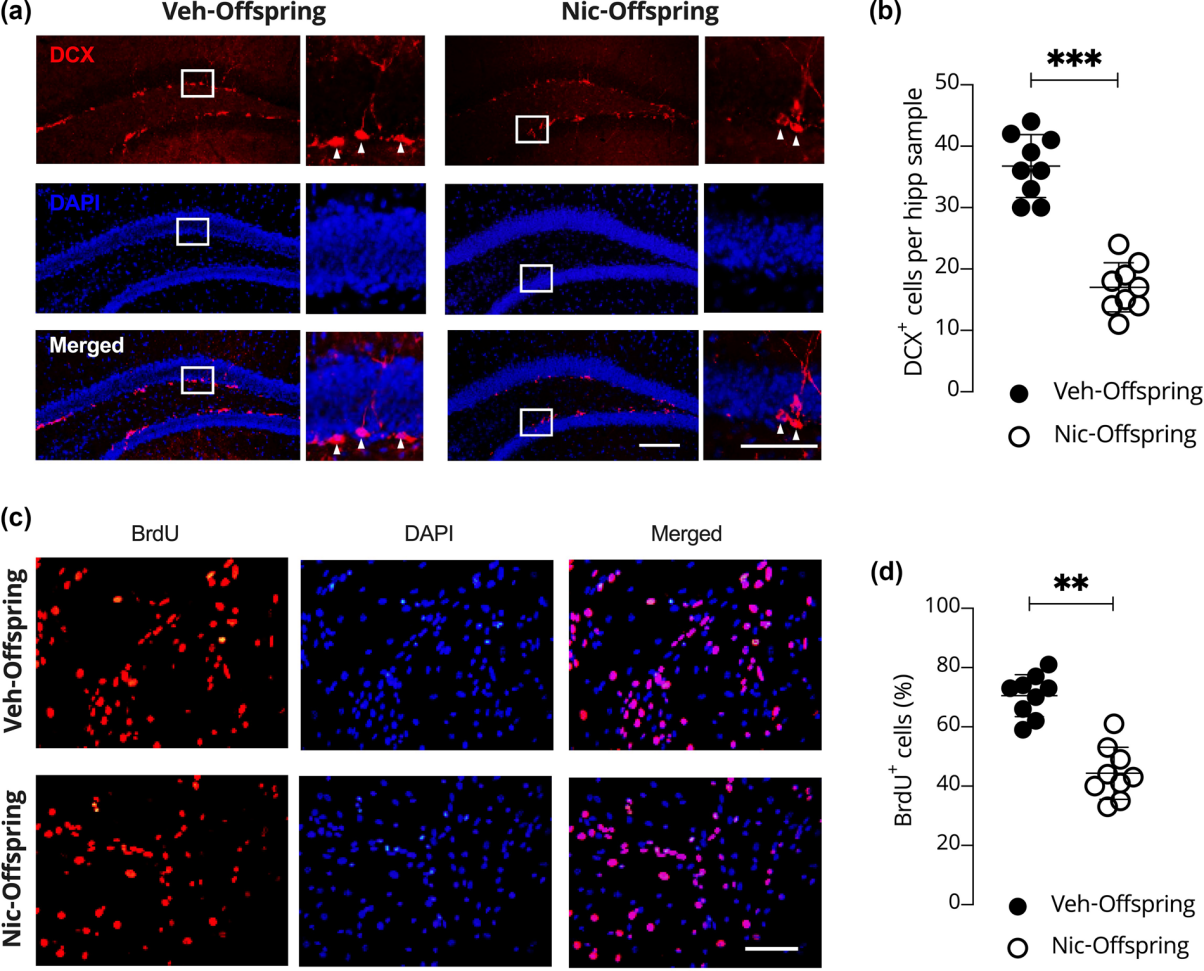
In vivo and in vitro evaluation of neurogenesis in male offspring from nicotine‐intoxicated maternal mice in comparison to normal offspring. (A) Expression of doublecortin (DCX)^+^ in cells of offspring from control (veh‐offspring) and nicotine‐intoxicated (nic‐offspring) dams. (B) Quantitative analysis of the number of DCX^+^ cells in both veh‐offspring and nic‐offspring. (C) In vitro BrdU incorporation in veh‐offspring and nic‐offspring. (D) Quantitative analysis of the number of BrdU^+^ cells in both veh‐offspring and nic‐offspring. Data are presented as means ± SD. One‐way ANOVAs were used to evaluate differences. **p < .01 and ***p < .001 compared to veh‐offspring. DAPI, 4′,6‐diamidino‐2‐phenylindole.

We evaluated the effect of maternal nicotine intoxication on neurogenesis and neural viability in the hippocampus of the offspring. DCX immunofluorescence staining showed a difference in the number of DCX^+^ cells between nic‐offspring (21.3 ± 1.8) and veh‐offspring (38.1 ± 2.1) (*p* < .001; Figure 5A,B). These results suggest that maternal nicotine intoxication attenuates the ability of offspring hippocampal neural stem cells to differentiate into neurons.

Overall, our findings suggest that offspring mice from nicotine‐intoxicated dams exhibited cognitive impairment and depression‐like behaviors, and the proliferation and differentiation of neural stem cells in the hippocampus were attenuated. To clarify the effect of maternal nicotine intoxication on hippocampal neurons, we cultured neural stem cells derived from both neonatal control and nicotine‐intoxicated dam offspring. Immunofluorescence staining using BrdU showed that the BrdU^+^ rate in the nic‐offspring group was 43.9% ± 6.8%, while in the normal group it was significantly higher at 69.0% ± 5.3% (*p* < .001; Figure 5B,C). These results show that offspring mice from nicotine‐intoxicated dams are more likely to have a lower rate of neural viability at different stages of brain development.

## 4 DISCUSSION

The primary objective of our study was to determine whether maternal nicotine intoxication elicited a stress response in male offspring mice and to further evaluate neurobiological changes. We only used male offspring mice in the behavioral experiments, as they did not have estrous cycles. Estrous cycles of female offspring could have negative effects on the experimental results (Wei et al., 2018). It has also been reported that nicotine addiction induces many psychiatric disorders, especially depression (Balfour & Ridley, 2000; Kutlu et al., 2015; Mineur & Picciotto, 2010). Besides, a depressive parent would have offspring with depressive‐like behaviors and cognitive deficits (Wei et al., 2018). Based on the above two facts, we hypothesized that maternal nicotine exposure results in depressive‐ and anxiety‐like behavior in offspring. In this current study, we verified our hypothesis and further tried to discover the underlying mechanism.

Briefly, our results showed that male offspring from nicotine‐intoxicated maternal mice showed a significant reduction in sucrose preference, and locomotor and exploratory activities. Offspring subjected to pregestational nicotine intoxication displayed enhanced depression‐ and anxiety‐like behaviors. These animals also exhibited impairments in recognition memory in the MWM test. Markers of HPA axis responsiveness and the monoaminergic system were significantly altered in pregestationally nicotine‐stressed offspring. The expression levels of *Bdnf*, *pCreb*, and *Sert* were also altered in the hippocampus and prefrontal cortex; however, there was no effect on *Creb* gene expression. In addition, neurogenesis and neural viability were impaired in the nicotine group compared to the control group.

Although the effect of maternal nicotine exposure on the behavioral outcomes of offspring has been documented in some studies, the effects of maternal nicotine exposure on adolescent behavior of male offspring and neurobiological traits have not been fully elucidated. Here, we investigated the effects of maternal nicotine exposure on adolescent behavioral outcomes and hippocampal neurogenesis. Smoking during pregnancy has been linked to stunted development in infants born to smokers (England et al., 2001). Prenatal nicotine exposure has also been linked to the development of attention deficit hyperactivity disorder, anxiety, and depression‐like symptoms in adult rats, corroborating the findings of earlier investigations (Alkam et al., 2013).

Across all phases of pregnancy and lactation, our results showed that maternal nicotine intoxication reduced learning and memory in male offspring, and increased depressive‐ and anxiety‐like behaviors. Open field evaluation was used to document the animals’ horizontal locomotor activity. Offspring of nicotine‐intoxicated dams showed decreased mobility time in the FST and spent less time in the center zone of the open field than did normal offspring. These findings are similar to another study showing that prenatal nicotine exposure in rodents results in postnatal hyperactivity, cognitive impairment, increased anxiety, somatosensory deficits, persistent neurochemical alterations, changes in sensitivity to nicotine, changes in nicotine self‐administration, and altered patterns of neural cell survival and synaptogenesis (Dwyer et al., 2009).

Nicotine‐exposed female mice showed a trend toward fewer live births, although this finding was not statistically significant. The weight gain rates were similar. However, offspring in the nicotine group exhibited delayed development in both the early physical development measure and the early neurobehavioral developmental index. For example, offspring from dams exposed to nicotine had considerably slower reflex righting times than those exposed to vehicle. Prenatal and early postnatal nicotine exposure disrupts neuronal development in the limbic and cortical regions, resulting in impaired mood and cognitive performance (Alkam et al., 2013). These results indicate that the children of nicotine users may experience a delay in the earliest stages of brain development.

Two major neurotransmitter systems, serotonin and norepinephrine, are implicated in hippocampus‐dependent memory (Björkqvist, 2001; Koolhaas et al., 1997). The hippocampus, hypothalamus, and prefrontal cortex are all parts of the brain implicated in the stress response among the most important regions in regulating depression (Mizoguchi et al., 2003). The children of mothers intoxicated with nicotine showed lower levels of serotonin in the plasma compared to controls, and higher levels of norepinephrine exclusively in the plasma. There is substantial evidence that the norepinephrine and serotonin neurotransmitter systems are dysregulated in depressive and anxious individuals, with the majority of the evidence favoring activation of the serotonin system and complex dysfunction of the norepinephrine system (most results indicate overactivation of this system) (Lee et al., 2007). In contrast, the dopaminergic system in the brain plays a significant role in regulating social behaviors in experimental animals (Cabib et al., 2000). Dopamine metabolism in the mesencephalic cortex and the border zones is enhanced after defeat in rats and mice (Mos & Van Valkenburg, 1979). This shift was also supported by our experimental findings. Specifically, we observed that the HPA axis, the monoaminergic system, and the expression of *Creb* and *Bdnf* transcription factors were all affected by nicotine in vivo. This was consistent with the results reported by Li et al. (2010). The activated form of CREB (pCREB) may act on the promoters (so‐called fourth messengers) of target genes (e.g., *Bdnf*, prodynorphin, *Pdyn*, *Fos*, and corticotropin‐releasing factor [*Crf*]) via its cognate gene regulatory element, the cAMP response element, thereby causing or mediating subsequent physiological responses (Gass & Riva, 2007).

Prenatal nicotine exposure has been linked to a variety of neurological abnormalities in offspring including attention deficit hyperactivity disorder, learning disabilities, and memory loss. Hippocampal neurogenesis encompasses several processes including neuronal maturation, synaptogenesis, and synaptic stability (Wang & Gondré‐Lewis, 2013). New neuron production, maturation, and integration in the adult hippocampus are essential for proper hippocampal functions, including learning and memory. In this study, we cultured neural stem cells derived from the offspring of nicotine‐intoxicated dams to evaluate neurogenesis. Notably, compared with the control group, the number of newly formed neurons in the brain networks of male offspring from nicotine‐intoxicated dams was significantly lower than the controls.

## 5 CONCLUSION

The results of this study show that maternal exposure to nicotine reduces offspring cognitive abilities, neurogenesis in the hippocampus, and microglial activation. These findings lend credence to the hypothesis that prenatal nicotine exposure has deleterious effects on children and interferes with normal neurogenetic processes in the offspring brain, resulting in behavioral and cognitive impairments.

## AUTHOR CONTRIBUTIONS

Linglong Deng designed the study and performed all experiments. Qing Wang and Ying Lou provided essential assistance. All authors reviewed and approved the final manuscript.

## CONFLICT OF INTEREST STATEMENT

The authors declare no conflicts of interest.

### PEER REVIEW

The peer review history for this article is available at https://publons.com/publon/10.1002/brb3.3052

## Data Availability

The datasets generated for this study are available on reasonable request to the corresponding author.
